# Reduction of Panton-Valentine Leukocidin Production in the Staphylococcal Strain USA300 After In Vitro Ascorbic Acid and Nicotinamide Treatment

**DOI:** 10.7759/cureus.47588

**Published:** 2023-10-24

**Authors:** Abdullah AlSaleh, Mohammad Shahid, Eman Farid, Khalid M Bindayna

**Affiliations:** 1 Microbiology, Immunology and Infectious Diseases, Arabian Gulf University, Manama, BHR; 2 Pathology/Immunology, Salmaniya Medical Complex, Ministry of Health, College of Medicine, Arabian Gulf University, Manama, BHR; 3 Microbiology, Arabian Gulf University, Manama, BHR

**Keywords:** elisa, usa300, pvl, nicotinamide, ascorbic acid

## Abstract

Background

Panton-Valentine leukocidin (PVL) is one of the most important determinants of virulence in *Staphylococcus aureus.* It is associated with a propensity for complicating skin and soft tissue infections and necrotizing pneumonia. This study aims to quantitively examine the effect of ascorbic acid and nicotinamide on PVL production in the reference strain USA300.

Methodology

Sandwich enzyme-linked immunosorbent assay (ELISA) was used to quantitively measure the production of PVL via the commercial LukS sandwich ELISA kit (IBT Bio-services, MD, USA).

Results

Incubating USA300 with subinhibitory concentrations of antioxidants resulted in a statistically significant eight-fold reduction in PVL production at 1.25 mg/mL and 30 mg/mL for ascorbic acid and nicotinamide, respectively. Although the mechanism by which antioxidants inhibit PVL production is yet to be elucidated, we suggest that it can be due to interrupting PVL gene expression.

Conclusions

Ascorbic acid and nicotinamide have the potential to be toxin-suppressing agents that may be effective in supporting the bactericidal effect of antibiotics to improve the outcome of PVL-associated infections; however, further extensive research is required.

## Introduction

*Staphylococcus aureus* is one of the most clinically challenging pathogens whose pathogenicity is attributed to evolutionary adaptations in persistence, virulence, and antibiotic resistance [1]. The prolific expression of exotoxins is a major contributor to its virulence [2]. Upon release, these exotoxins continuously damage the host cells and tissues independently from the bacteria, which enables further bacterial dissemination, complicating the clinical management of staphylococcal infections [2]. Arguably, one of the most important determinants of virulence in *S. aureus* is Panton-Valentine leukocidin (PVL), a bi-component cytotoxin that is frequently detected in clinical settings [3]. It is associated with complicating skin and soft tissue infections (SSTIs) and necrotizing pneumonia, as well as implicated in increased disease severity in young adults and healthy children [4]. PVL targets the membrane of immune cells, mainly neutrophils, where oligomerization and the pore formation mechanism ensue, resulting in perforating the cell membrane and inducing cell death as well as the release of proinflammatory markers [5,6].

Incorporating toxin-suppressing agents into the clinical management of staphylococcal infections has been suggested. Indeed, protein synthesis inhibitors (e.g., clindamycin and linezolid) as well as intravenous immunoglobulins (IVIgs) have been tested in the treatment of severe PVL-associated infections with promising results [7-9]. Nevertheless, the potential toxicity derived from antibiotic use and the high cost of IVIgs present a sizable setback in managing these infections. Fortunately, antioxidants (e.g., vitamins B, C, and E) have shown promise in interrupting toxin expression and downregulating virulence genes against bacterial superbugs [10-13]. In our previous work, we managed to illustrate the inhibitory effect of ascorbic acid and nicotinamide on PVL cytolytic activity [14]. Thus, in this study, we aim to quantitively examine the effect of ascorbic acid and nicotinamide, individually, on PVL production in the reference strain USA300.

## Materials and methods

Minimum inhibitory concentration (MIC)

A two-fold serial microdilution was prepared in Mueller-Hinton broth (Sigma-Aldrich, St. Louis, MO, USA) for each agent in accordance with Clinical and Laboratory Standards Institute (CLSI) (M07) guidelines [15]. The inoculum used was the PVL-producing methicillin-resistant *Staphylococcus aureus* (MRSA) reference strain USA300, an epidemic PVL-producing community-acquired MRSA strain.

PVL induction

Inducing the production of PVL was facilitated by incubating the bacteria in Lauria-Bertani (LB) broth (Sigma-Aldrich, St. Louis, MO, USA) with 1/4 MIC of oxacillin (12 µg/mL) for 24 hours at 37°C with vigorous shaking, as mentioned by Dumitrescu et al. [16].

Antioxidant treatment

The effect of antioxidants on PVL production was tested by the addition of ascorbic acid (1.25 mg/mL and 0.3 mg/mL) and nicotinamide (30 mg/mL and 7.5 mg/mL) individually to the culture media. These concentrations represent 1/2 and 1/8 of the MIC of each agent.

PVL yield

Collecting the produced PVL from the supernatant was performed in accordance with the methodology mentioned by Badiou et al. [17]. Colonies of overnight USA300 culture (1 x 10^6^ CFU/mL) were transferred into 5 mL of media (LB broth + 12 µg/mL of oxacillin). Antioxidants could be added to the culture media depending on the assay, followed by incubation for 18 hours at 37°C with vigorous shaking. After incubation, the cultures were centrifuged at 2,800 rpm for 10 minutes at 4°C, the tubes were sterilized for 60 minutes by heating at 95°C, followed by cooling on ice for five minutes, and reserved in the fridge (4°C) until needed.

Sandwich enzyme-linked immunosorbent assay (ELISA)

PVL detection was performed via sandwich ELISA using the commercial LukS sandwich ELISA kit (IBT Bio-services, MD, USA). The process was conducted in accordance with the datasheet provided by the manufacturer. The absorbance was measured via a FLUOstar® OMEGA Plate Reader (BMG LABTECH, Ortenberg, Germany) at 650 nm wavelength. A standard curve (4PL) was created to determine the concentration of the unknown samples using the following equation: X = C × [(A-Y)/(Y-D)^(1/B), where X = ng/mL of LukS, Y = absorbance value (650 nm), A = lower asymptote, B = slope, C = infliction point, and D = upper asymptote.

Statistical analysis

Calculations and statistical analysis were conducted using Microsoft Excel 365 (Microsoft Corp., Redmond, WA, USA). All experiments were performed in triplicates and data were presented as mean and SD whenever applicable. A t-test was used to determine statistically significant differences between untreated and antioxidant-treated assays. Differences between values were considered significant at p-values <0.001. The 4PL curve was formulated using MyAssays Data Analysis Tool (MyAssays Ltd, USA).

## Results

MIC

A two-fold serial microdilution was used to determine the MIC of each agent in accordance with CLSI M07 guidelines [15]. MIC values are recorded in Table 1.

**Table 1:** MIC values for the tested agents against USA300. MIC = minimum inhibitory concentration

Agent	MIC
Ascorbic acid	2.5 mg/mL
Nicotinamide	60 mg/mL
Oxacillin	48 mg/mL

Quantification of PVL

**Figure 1:**
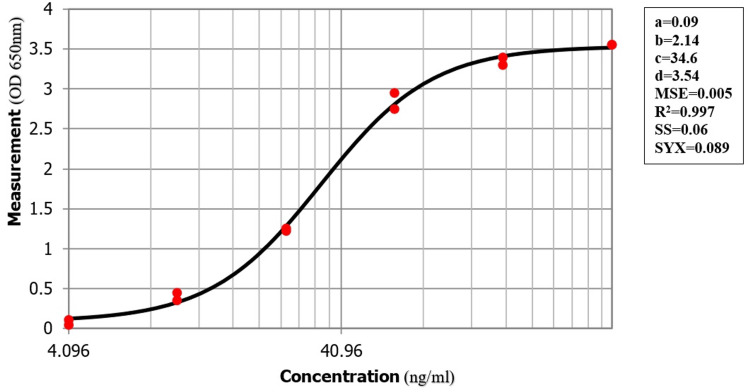
The 4PL standard curve stemming from LukS quantitative enzyme-linked immunosorbent assay.

PVL production in USA300 was quantified via the commercial LukS sandwich ELISA kit. The standard 4PL curve (Figure 1) was used to measure PVL production in USA300. Without inducing PVL production by adding 12 µg/mL oxacillin, the kit could not detect any target protein in all antioxidant treatments, as seen in Table 2. However, with induction, 16 ng/mL was measured as the concentration of PVL produced by USA300 without antioxidant treatment. Incubating USA300 with subinhibitory concentrations of antioxidants resulted in a statistically significant eight-fold reduction of PVL production at 1.25 mg/mL and 30 mg/mL for ascorbic acid and nicotinamide, respectively (Table 2). As shown in Table 2, PVL concentration was reduced to 2 ng/mL when incubating the bacteria with 1.25 mg/mL of ascorbic acid. Consequently, at a lower concentration of ascorbic acid (0.3 mg/mL), the measured PVL concentration increased to 13 ng/mL. A similar dose-dependent reduction was illustrated with the nicotinamide assay, as measurements of produced PVL decreased to 2 ng/mL at 30 mg/mL of nicotinamide incubation and increased to 10 ng/mL when incubated with 7.5 mg/mL of nicotinamide.

**Table 2:** Concentration of PVL produced by USA300 under antioxidant treatment. All treatments were performed in triplicates and are presented as mean ± SD. A t-test was used to determine the statistically significant differences between untreated and antioxidant-treated assays. P-values <0.001 are statistically significant. AA = ascorbic acid; LB = Luria-Bertani; NAM = nicotinamide; OD = optical density; OX = oxacillin; PVL = Panton-Valentine leukocidin; SD = standard deviation

Treatment		OD (650 nm), mean ± SD	PVL concentration	P-value
Not induced	Bacteria + LB broth only	0.04 (	Not determined	-
	+1.25 mg/mL AA	0.04 (	Not determined	-
	+0.3 mg/mL AA	0.04 (	Not determined	-
	+30 mg/mL NAM	0.04 (	Not determined	-
	+7.5 mg/mL NAM	0.04 (	Not determined	-
Induced	+12 µg/mL OX	0.6 ± 0.2	16 ng/mL	-
	+12 µg/mL OX + 1.25 mg/mL AA	0.08 ± 0	2 ng/mL	<0.001
	+12 µg/mL OX + 0.3 mg/mL AA	0.5 ± 0.5	13 ng/mL	>0.001
	+12 µg/mL OX + 30 mg/mL NAM	0.08 ± 0.1	2 ng/mL	<0.001
	+12 µg/mL OX + 7.5 mg/mL NAM	0.4 ± 0.5	10 ng/mL	>0.001

## Discussion

PVL is a cytolytic exotoxin produced by *S. aureus* [18]. It is a bi-component cytotoxin that mainly targets polymorphonuclear cells and is associated with conditions ranging from primary SSTIs to severe necrotizing pneumonia [19,20]. It has been suggested that managing PVL-associated infections should not be predicated on eradicating the bacteria only but also on neutralizing and minimizing the synthesis and production of PVL, especially in severe cases [7].

In this study, the effect of subinhibitory concentrations of ascorbic acid and nicotinamide, individually, was investigated on PVL production in the reference strain USA300. A significant reduction in PVL concentrations was achieved when incubating USA300 with subinhibitory concentrations of the aforementioned antioxidants. Indeed, PVL concentration was reduced from 16 ng/mL to 2 ng/mL, constituting a statistically significant eight-fold reduction in PVL production after incubating USA300 with 1.25 mg/mL of ascorbic acid and 30 mg/mL of nicotinamide (Table 2).

Targeting the expression of virulence agents such as PVL when managing bacterial infection has been illustrated before. Incubating PVL-producing strains with subinhibitory concentrations of antibiotics that function as RNA and protein synthesis inhibitors against staphylococcal pathogens such as linezolid, clindamycin, fusidic acid, rifampicin, and tigecycline has resulted in a significant reduction in PVL mRNA levels and protein expression [16,21,22]. Interestingly, utilizing this inhibitory effect in managing staphylococcal infections has been linked to better clinical outcomes. In a necrotizing pneumonia rabbit model, early administration of linezolid inhibited PVL production and improved survival rates by four times more in the vancomycin-only therapy group [23]. Furthermore, several case reports have suggested that the early administration of toxin-suppressing agents such as linezolid or clindamycin may improve the clinical outcomes of PVL-associated necrotizing pneumonia [8]. Furthermore, inactivating PVL production has been shown to reduce pathology and bacterial count in infected mouse corneas and human-cultured corneal epithelial cells [9].

To our knowledge, as the findings presented in this study have never been investigated before, the modality by which PVL production is inhibited by antioxidants is yet to be elucidated. Nevertheless, the effect of antioxidants on the expression of virulence factors other than PVL has been explored before and may explain the findings in this study. Indeed, ascorbic acid has shown a remarkable ability to interrupt the expression of bacterial virulence factors such as biofilm and capsule formation proteins and exopolysaccharides in *Bacillus subtilis*, *Escherichia coli*, *Klebsiella pneumoniae*, and *Pseudomonas aeruginosa* [11,12,24,25]. Similarly, nicotinamide has been shown to inhibit protein synthesis in *Mycobacterium tuberculosis* and the biofilm formation of *Streptococcus mutans* [13,26]. Therefore, we suggest that ascorbic acid and nicotinamide may interrupt the bacterial protein expression mechanism, entirely or partially; however, more investigations are needed.

Limitations

One major limitation of this study is not including PVL-producing MRSA clinical isolates owing to the limited supply of the LukS ELISA kit. Unfortunately, we were unable to obtain a similar kit from another supplier.

## Conclusions

This study illustrated a significant reduction in PVL produced by USA300 when incubated with ascorbic acid and nicotinamide individually. This finding is significant because an integral part of managing staphylococcal infections is to inhibit the production of virulence factors such as toxins and ultimately block their biological effect. We presented potential toxin-suppressing agents, i.e., ascorbic acid and nicotinamide, that may be effective in supporting the bactericidal effect of antibiotics to improve the outcome of PVL-associated infections.
